# Spatialization of Time in Mian

**DOI:** 10.3389/fpsyg.2012.00485

**Published:** 2012-11-19

**Authors:** Sebastian Fedden, Lera Boroditsky

**Affiliations:** ^1^University of SurreyGuildford, UK; ^2^Max Planck Institute for PsycholinguisticsNijmegen, Netherlands; ^3^Stanford UniversityPalo Alto, CA, USA

**Keywords:** space, time, Mian, Papuan, river-based spatial system

## Abstract

We examine representations of time among the Mianmin of Papua New Guinea. We begin by describing the patterns of spatial and temporal reference in Mian. Mian uses a system of spatial terms that derive from the orientation and direction of the Hak and Sek rivers and the surrounding landscape. We then report results from a temporal arrangement task administered to a group of Mian speakers. The results reveal evidence for a variety of temporal representations. Some participants arranged time with respect to their bodies (left to right or toward the body). Others arranged time as laid out on the landscape, roughly along the east/west axis (either east to west or west to east). This absolute pattern is consistent both with the axis of the motion of the sun and the orientation of the two rivers, which provides the basis for spatial reference in the Mian language. The results also suggest an increase in left to right temporal representations with increasing years of formal education (and the reverse pattern for absolute spatial representations for time). These results extend previous work on spatial representations for time to a new geographical region, physical environment, and linguistic and cultural system.

## Introduction

People around the world rely on space to represent time. We spatialize time in language and gesture, as well as in graphs, time-lines, clocks, sundials, and calendars. However, the particular ways that time is spatialized differ across languages and cultures. Previous work suggests that the way people spatialize time depends in part on the set of spatial representations and reference frames that are available in the linguistic or cultural environment (Boroditsky and Gaby, 2010; Núñez et al., 2012).

Languages differ in how they typically describe and partition space, and in how an object (the figure) is typically located with respect to another object (the ground). Levinson (1996, 2003; pp 38–50) distinguishes three basic frames of reference: absolute, relative, and intrinsic (also see Tenbrink and Kuhn, 2011). The absolute frame of reference involves fixed directions, which define the coordinate system and which are independent of figure, ground, or perceiver; examples of such fixed directions are compass bearings or landscape features like rivers or coastlines, e.g., *The school is north of the hospital* or *The school is upriver of the hospital*. In the relative frame of reference the coordinate system originates in a viewpoint, which is the location of the perceiver of figure and ground, e.g., *The school is to the left of the hospital* (as seen from the perspective of the perceiver). The intrinsic frame of reference uses an object-centered coordinate system whose orientation is determined by intrinsic or inherent properties of the ground, e.g., *The tree is in front of the school* (being the side of the building with the main entrance).

Previous work has shown that people whose language prominently uses absolute frames of reference to represent space, may also come to spatialize time in absolute space. For example, the Kuuk Thaayorre and Wik Mungkan speakers from Cape York in Australia rely on landscape-based cardinal directions to talk about space and also tend to lay out time as proceeding from east to west (Boroditsky and Gaby, 2010). By measuring the pointing direction of naturally produced gestures Núñez et al. (2012) show that the Yupno of Papua New Guinea, whose language makes extensive use of the absolute terms “uphill” and “downhill,” construe the past as downhill, the future as uphill, and the present moment as being in the same location as the speaker.

These patterns are strikingly different from what we find in English speakers, who commonly rely on body-centered relative terms like “left” and “right” to specify relations in space, and lay out time as proceeding from left to right in body-centered coordinates.

In this paper we examine representations of time among the Mianmin of Papua New Guinea. We begin by broadly describing the patterns of spatial and temporal reference in Mian, which differ in many fascinating ways from English. We then focus on spatial frames of reference. Mian employs an absolute frame of reference, which is associated with the lay of the Hak and Sek rivers, running roughly parallel near the Mianmin village. This absolute frame is invoked with the terms “upriver” and “downriver.”

As an initial investigation into how the Mianmin represent time we present results from a non-linguistic temporal ordering task in which participants are asked to arrange picture sequences on the ground (e.g., pictures of a man at different ages) to demonstrate the temporal order implied in the pictures. This task is adapted from the one administered to Kuuk Thayorre speakers by Boroditsky and Gaby (2010). This task allows us to examine one of the conceptual differences suggested by patterns in language: the reliance on absolute spatial frames of reference in representing time. We examine whether patterns in language are reflected in people’s spatializations of time in this non-linguistic temporal representation task and further analyze people’s spatializations for time as a function of age, education, and literacy.

The interest of the Mian language is that its absolute system is different from the one found in Kuuk Thayorre in that it relies on landmarks (namely rivers) rather than cardinal directions. But if dominant frames of reference have an impact on the way humans represent time we would expect to find that Mian speakers arranged temporal sequences in space in alignment with the course of the rivers. So the research question arising from our knowledge about absolute representation of time in Kuuk Thayorre is whether the prominence of the Mianmin river system in spatial reference might also be reflected in Mian representations of time. The results of the present study – albeit preliminary – suggest that this is indeed the case.

## Description of Mian

The topic of this section is the spatial and temporal language of the Papuan language Mian (Fedden, 2007, 2011), a member of the Mountain Ok branch within the Ok family of languages (Healey, 1964; Voorhoeve, 2005), which belongs to the Trans-New Guinea (TNG) family (cf. Wurm, 1982; Pawley, 2005; Ross, 2005). To provide appropriate context, we begin with a description of the Mian linguistic community, and continue with a broad survey of temporal and spatial reference in Mian.

Mian is spoken in Telefomin District of Sandaun Province in Papua New Guinea. The language has about 1,400 speakers according to the 2000 census (Lewis, 2009). The data presented here are based on the eastern dialect. Most Mian speakers under the age of 75 also speak the New Guinea-variety of Neo-Melanesian Pidgin, Tok Pisin, and older male speakers (above 50 years) also speak – or at least understand – the closely related neighboring language Telefol. Tifal or other Ok languages are not known among Mian speakers. English is becoming more and more important. The school years 3–12 are taught almost entirely in English and a good command of English is essential for those who want to escape the traditional life of a subsistence farmer and obtain a better position outside the village.

The Mian-speaking community uses a practical orthography, developed by Smith and Weston (1974). This orthography is based on the Latin alphabet and written from left to right. Most people are literate in Mian and Tok Pisin. While reading materials in Mian are limited there are a few readers in the language and a translation of the New Testament (Smith and Weston, 1986). The latter publication is used widely within the community. Some older speakers, who have not learned the Mian orthography, do not write Mian but still write Tok Pisin, if they speak it. Written communication with Telefol speakers is in Tok Pisin. Younger speakers are also literate in English.

Geographically, the Mianmin area is delimited by the August and May rivers in the west and east, respectively, and the Hindenburg Range in the south. This area is roughly located between the 141st and 142nd degrees of longitude and between the 4th and 5th parallels. There are peaks ranging from 1,000 to 2,800 m throughout the area. The landscape is characterized by hills and mountains covered by primary and secondary rainforest and a tangle of rivers. There are no roads, only paths, and flying and walking are the only means of getting around.

The Mianmin practice swidden (slash-and-mulch) agriculture. Their starch staple is taro (*Colocasia esculenta*) and they supplement their diet with hunted game, mainly pigs (*Sus scrofa*). Dietary cannibalism was practiced in pre-colonial times^1^.

### Spatial reference

Mian uses intrinsic, relative, and absolute frames of reference to locate a figure with respect to the ground. There are no words for “left” or “right” in the language. The following nominals do exist however:

**Table:** 

(1)	*kweital* “right hand; correct; first-born of twins”
	*afan* “left hand; wrong, strange, weird; second-born of twins”

Reference to space can be done intrinsically with complex spatial expressions like (the backside of a tree is the side leaning toward the ground):

**Table:** 

(2)	*as* = *e*	*abuksin* = *daa*
	tree = SG.N1	back = LOC
	*mâa’-bi-Ø-ebo* = *be*	
	stand_up.PFV-AUX.IPFV-IPFV-2SG.SBJ = DECL	
	“You’re standing at the back of the tree.”	

This is the opposite of what one finds in Chamus (a Nilo-Saharan language of Kenya), where the inclined side is treated as the front (Heine, 1997; p. 13)^2^.

The nouns *kweital* “right hand” and *afan* “left hand” can be used intrinsically to locate a figure at the right- or left-hand side of a human ground, while back and front can be used with all kinds of grounds. While the English spatial terms *back* and *front* can be used relatively or intrinsically (Levinson, 2003; p. 31) the Mian terms *abuksin* “back(side)” and *kibikibasin* “front(side) [<*kibi* face]” can only be used intrinsically. Intrinsic terms are only used in specific locally restricted situations.

Mian does not have lexemes for cardinal directions. Absolute reference to space with respect to the horizontal dimension is done with the spatial terms given in (3):

**Table:** 

(3)	*met* “upriver”
	*tab* “downriver”
	*tām* “sideways of the river”

**Figure 1:**
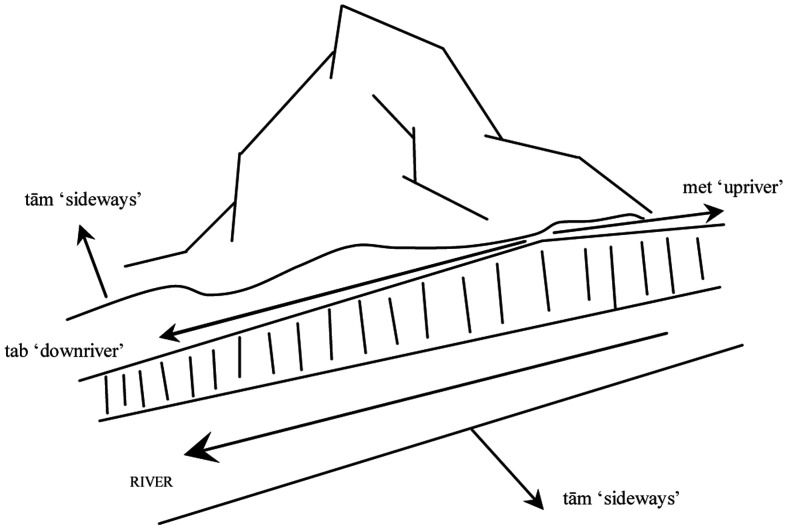
**Mian absolute terms and the topographic environment**.

These spatial terms are intimately linked to the topographic environment in which the speakers of the language live. This is illustrated in Figure 1. (The vertical lines above the river indicate the steep slope leading down to the river bank).

The main axis of orientation for the absolute frame of reference is the orientation of the two rivers Hak and Sek, which run roughly parallel near Mianmin. The terms *met* “upriver” and *tab* “downriver” can either have a locative or an allative meaning. Examples are given in (4) and (5):

**Table:** 

(4)	*skul*	*am*	*met*
	school	house	upriver
	“upriver at/to the school house”		

**Table:** 

(5)	*Skiobib*	*tab*
	PN	downriver
	“downriver at/to Skiobib”	

These two terms refer to fixed directions provided by the course of the rivers near Mianmin. In the examples (4) and (5) above, they could also be used in this way if the school house or the place called Skiobib were not situated at or close to the river. They are not restricted to direct references to a location at the river. The system is an abstraction from an environmental gradient (cf. Levinson, 2003; p. 48), in this case the river.

The directional *tām* “sideways” refers to any direction or location sideways of the river:

**Table:** 

(6)	*Asuneb* = *e*	*am*	*tām*
	PN = SG.M	house	sideways
	“sideways (of the river) at/to A.’s house”	

The absolute terms *met* “upriver,” *tab* “downriver,” and *tām* “sideways of the river” as means of referring to directions and location on the horizontal plane are ubiquitous in spoken Mian. In fact, they are the only terms available for the reference to these directions.

As is typical for a Papuan language, Mian has terms for “up,” “down” for reference to the vertical dimension and a term for “across.” These three terms are given in (7). None of them can be combined with each other or with the terms in (3) above.

**Table:** 

(7)	*ut*	“up(ward)”
	*daak*	“down(ward)”
	*wāt*	“across”

These form a single discrete word class together with the terms *met* “upriver,” *tab* “downriver,” and *tām* “sideways” called directionals in Fedden (2011; pp 140–143), which have the following distinctive features: (i) They can be used as adverbs, e.g., *daak un-Ø-e* = *be* [down(ward) go.PFV-REAL-3SG.M.SBJ = DECL] “he went down,” (ii) as postpositions (examples will be given further below) or (iii) as intransitive verbs of motion when inflected directly:

**Table:** 

(8)	*met-n-i* = *a*
	upriver-SS.DEQ-1SG.SBJ = MED
	“I go upriver and then I …”

Directionals are highly frequent items and ubiquitous in Mian discourse. Most clauses with a motion verb also contain a directional.

The terms *ut* “up(ward)” and *daak* “down(ward)” are used to refer to the vertical dimension:

**Table:** 

(9)	*ut-n-ib* = *a*	*Sek*
	up(ward)-SS.SEQ-2/3PL.AN.SBJ = MED	PN
	*tibín*	*ut*
	river_head	up
	“they went up, up to the river head of the river Sek”	

**Table:** 

(10)	*Milsen* = *e*	*bib*	*daak*
	PN = SG.M	village	down
	*bi-Ø-e* = *be*		
	be.there-IPFV-3SG.M.SBJ = DECL		
	“M. is down at/in the village.”		

The terms *ut* “up(ward)” and *daak* “down(ward)” are absolute in the sense that the vertical dimension is determined by gravity. However, when referring to the vertical dimension, the relative viewpoint (i.e., everything above the speaker vs. everything below the speaker) and the absolute gravitational field typically align (Levinson, 2003; p. 75).

*Wāt* “across” is not an absolute term. It is used for a trajectory traversing a salient axis, for example a valley or river:

**Table:** 

(11)	*Hak*	*taman*	*wāt*
	PN	valley	across
	“across the Hak river valley”		

In large(r)-scale contexts the directionals *w*ā*t* and *daak* have a different sense. They can be used to refer to far-away and very far-away places, for instance places as far-away as Vanimo (25 km), Port Moresby (roughly 800 km), or Australia, all of which are *w*ā*t*. There is some inter-speaker variation so generalizations are hard to make (e.g., some use *daak* for Australia as well) but it seems that far-away places like Port Moresby (about whose distance speakers have fairly accurate knowledge) are generally *w*ā*t*, while very far-away places like Europe (about whose distance speakers do not have accurate knowledge) are *daak*. None of the other directionals, are used outside the local scale.

Directionals can be employed in small(er)-scale environments, in which *met* and *tab* are not used with reference to the river as a landmark but where *met* refers to a location near the speaker, while *tab* refers to a location away from the speaker. In this context the terms *met* and *tab* are not used absolutely, but it seems that the upstream-downstream feature of the river can be extended to an imaginary axis between two participants. Metaphorically speaking, the “river” flows away from the speaker and toward the addressee:

**Table:** 

(12)	*futblông* = *e*	*kēb* = *daa*
	cigarette_box = SG.N1	2SG.M = LOC
	*tab*	
	away.from.speaker	
	*o-fâ-n-ebo* = *be*
	3SG.O-put.PFV-REAL-2SG.SBJ = DECL	

“You put the cigarette box down(river) near you” (in a situation in which the “river”-axis between the participants was orthogonal to the actual river).

Two directionals are also extended to the location of certain body parts with respect to the vertical dimension. Locations around the upper part of the human body are commonly referred to as *ut* “up(ward),” in (13) and locations around the lower part as *tab* “down(ward),” in (14):

**Table:** 

(13)	*kwel*	*ut*
	neck	up(ward)
	“up at the neck”	

**Table:** 

(14)	*kakam*	*tab*
	buttocks	down(ward)
	“down at the buttocks”	

Note that here *ut* “up(ward)” is in opposition with *tab* “down(ward)” rather than *daak* “down(ward),” which is the complementary term to *ut* in geographical space. Clearly, we are not dealing with the “downriver”-sense of *tab* here since the direction of the river does not play a role in the interpretation of (14). In this case a different sense of *tab*, namely “down(ward)” is selected. *Tab* has the sense “downriver” in geographical space and “down(ward)” – in opposition to *ut* “up(ward)” – when referring to the vertical axis of the human body. It is cross-linguistically well-known that the same term can be used in environments of different scale (Levinson, 2003; p. 247).

The language has two other spatial postpositions, namely *dim* “on(to)” and *tem* “in.” These do not belong to the word class of directionals because they show different grammatical behavior, but they are nonetheless important items of the spatial vocabulary because they are metaphorically extended to express temporal concepts (see Temporal Reference below):

**Table:** 

(15)	*tebol*	*dim*
	table	on(to)
	“on(to) the table”	

**Table:** 

(16)	*smē*	*tem*
	cave	in
	“in the cave”	

Complex postpositions in Mian are compounds consisting of either *dim* “on(to)” or *tem* “in” and a directional, e.g., *temwât*, consisting of *tem* “in” and *wāt* “across” with the compositional meaning “across in(to)”:

**Table:** 

(17)	*kwéit* = *e*	*tem-wāt*
	sugarcane = SG.N1	into-across
	*on-s-e* = *a*	
	go.PFV-DS.SEQ-3SG.M.SBJ = MED	
	“he went across into the sugar cane and then someone else…”	

Examples of other complex postpositions are given in (18).

**Table:** 

(18)	*dim-ut*	“up on(to)”
	*dim-daak*	“down on(to)”
	*dim-wāt*	“across on(to)”
	*tem-daak*	“down in(to)”
	*tem-tām*	“sideways in(to)”

After looking at the spatial repertory of Mian and showing how intricately it is linked to the local environment we will now deal briefly with temporal reference in the language.

### Temporal reference

While directionals are highly frequent in spatial reference, they barely show up in temporal reference. However, *tab* can be used to describe the passage of time. Presumably, this sense of *tab* is a metaphorical extension of the spatial “downriver”-sense of *tab* described above. An example is (19):

**Table:** 

(19)	*am* = *o*	*hebmamsâb*
	time = N2	quickly
	*tab*	*tl-Ø-o* = *be*
	down	come.PFV-REAL-N2.SBJ = DECL
	“The time passed quickly.”	

*Dim* “on(to)” is generally used to refer to points in time, e.g.:

**Table:** 

(20)	*Febluali* = *e*	*dim*	*ē-ta*
	PN = SG.N1	on	SG.N1-EMPH
	*imín*	*tl-aamab-i* = *be*
	again	come.PFV-IRR-1SG.SBJ = DECL	
	“I’ll come again in February.”		

*Tem* “in,” on the other hand, in a temporal sense is only found in a few postpositional phrases:

**Table:** 

(21)	*mikík*	*tem*
	beginning	in
	“in the beginning, at first”	

Of the complex postpositions only *temwât* with the spatial meaning “across into” can be used temporally with the meaning “while”:

**Table:** 

(22)	*ī*	*miting*	*ke-b-ib* = *o*
	they	meeting	do-IPFV-2/3PL.AN.SBJ = N2
	*temwât* = *o*		
	while = N2		
	“while they were holding a meeting…”		

For locating events in time temporal adverbials are used, none of which are transparently spatial in origin, with the exception of the first two in the following list:

**Table:** 

(23)	*ōlo* “now” (demonstrative pronoun *ōlo* “this”)
	*abuko* “later, afterward” (*abuk* “back”)
	*memâlo* “today, now” (*memâ* “new”)
	*sino* “formerly, before, earlier” (*sin* “old”)
	*sintalo* “yesterday”
	*sintalo ō sintao* “the day before yesterday” [lit. “yesterday it’s yesterday”]
	*sinanggwáno* “a very long time ago”
	*sinanggwánanomo* “in the far future”
	*kutimibo* “at night, in the early morning” (*kutimib* “night, early morning”)

In terms of morphological marking of tense distinctions, Mian has five deictic past tenses. These are (with a brief semantic characterization in brackets):

**Table:** 

(24)	*-nab* “Near past” (a few minutes ago)
	*-so* “Hesternal past” (yesterday and the day before yesterday)
	*-b^H^* “Non-hodernal past” (in the past, but not today)^3^
	*-bio* “General past” (from a few hours ago into the far past, excluding yesterday)
	*-s* “Remote past” (many years ago)

Realis forms commonly have past time reference as well, imperfective forms have present time reference unless there is an indication to the contrary, for example a temporal adverb with past time reference. Future time reference is a function of irrealis mood.

### The role of the sun in segmenting the phases of the day

Important and salient phases of the day are referred to by describing where the sun (*afók*) is at that particular time. With the advent of watches to keep track of the passage of time, these phrases seem to fall slowly into disuse. Examples are given below with the approximate time of the day they are used for:

**Table:** 

(25)	*afóko glit genota* “the sun is rising” (6:00–7:30 A.M.)
	*afóko umtlota* “the sun has almost cleared the mountains” (7:30–8:00 A.M.)
	*afóko tubunoa blatblat tlota* “the sun shines and her light becomes clear” (around 8:00 A.M.)
	*afóko tubunoa kelanota* “the sun is shining and going toward midday” (9:00–11:00 A.M.)
	*afóko isaak ut tlobe* “the sun has come up to midday position” (12:00 A.M.)
	*afóko tlaa delwabmaanota* “the sun is sinking” (1:00–6:00 P.M.)
	*takeib afók tubunota* “the sun is setting” (5:00–6:30 P.M.)

Grammatically, these are full clauses, each with the sun as the subject, which would be used to indicate a certain time or phase of the day. *Afók* is also the word for grandmother and, in fact, any female ancestor. While the Mianmin do not believe that humans were created by the sun, their mythical ancestor woman who created the first Mianmin came from the Highlands, i.e., from the east. This shows that the sun and its path plays an important part in talking about different phases of the day. It is therefore conceivable that the path of the sun is also important in the spatialization of temporal sequences.

### Other cultural representations of time

Nowadays, the western calendar is used and a few people have watches to keep track of time, while other kinds of clocks are absent. The word for time is *am*, which also means “day.” There are no personifications of Time.

In pre-colonial times, people counted months and days (i.e., moon and sun cycles)^4^. More precisely, they counted nights. They used knots in a vine to keep track of time or a body-part tally system. Counting in this system commences with pointing to or touching the thumb, followed by the fingers of the hand, then up the side of the body (wrist, forearm, elbow, shoulder joint, shoulder, cheek, ear, eye, nose) each time adding one so that one reaches 14 when touching the nose. From there, counting proceeds down the opposite side of the body (the pointing or touching is done with the other hand now) till the whole procedure ends with the little finger of the other hand and the number 27 (Fedden, 2011; pp 147–148). The body-part tally system and its role in keeping track of passing time is analyzed in detail in Fedden (2012).

## Non-Linguistic Temporal Reasoning Task

In this section we offer an initial investigation into how the Mianmin represent time outside the linguistic system. We focus on spatial frames of reference and ask whether the prominence of the Mianmin river system in spatial reference is reflected in Mian representations of time. We present results from a non-linguistic temporal ordering task in which participants are asked to arrange picture sequences on the ground (e.g., pictures of a man at different ages) to demonstrate the temporal order implied in the pictures. We were interested in whether patterns in spatial and temporal language might be reflected in how the Mianmin represent time in this non-linguistic task.

In addition to spatial frames of reference, many other aspects of language and culture may play a role in shaping temporal thinking. For example, writing direction is an important determinant of how people organize time (e.g., Tversky et al., 1991; Fuhrman and Boroditsky, 2010; Boroditsky et al., 2011), and formal schooling may be an important factor. This initial study with the Mianmin allows us to further examine representations of time in a community that is very different from the industrialized and formally schooled populations typically included in previous work. This allows us to ask not only how uniform representations of time might be across cultures, but also how uniform they might be within a community like the Mianmin who are exposed to many conflicting cultural influences. We examine whether how the Mianmin spatialize time differs as a function of age, education, and literacy.

### Participants and experimenter

Nine native Mian speakers aged 13–55 participated in the study (three Female). For all participants, Mian was their only native language. All also spoke Tok Pisin (Mean proficiency = 4.8 out of 5, as assessed by the experimenter), seven spoke some English (Mean proficiency = 2.6 out of 5) and five spoke some Telefol (Mean proficiency = 2.0 out of 5). All participants were to some degree literate in Mian and Tok Pisin (Mean literacy = 7.1 and 6.7 out of 10, respectively), and five of the participants were also literate in English (Mean literacy = 6.2 out of 10)^5^. The participants’ level of education spanned from having no formal education to completion of teacher’s college (Mean years = 7.44, SD = 4.75). It would have been desirable to have a larger and more homogenous group of participants, but this was unfortunately not possible to achieve due to the limited number of speakers we could draw on.

The experimenter (SF) has been working on Mian since 2003. He spent a total of 11 months in the field (distributed over three trips) and has working proficiency in the language.

### Task

Participants were tested on a temporal card arrangement task adapted from Boroditsky and Gaby (2010; see also Fuhrman and Boroditsky, 2010). On each trial participants were given a shuffled set of four picture cards and were asked to arrange the cards on the ground to show the correct order. The picture sets depicted simple temporal progressions (e.g., a man at different ages or an apple being eaten). Each participant was tested in two sittings with an average facing direction difference of 145 degrees between sittings (median and mode facing direction difference = 160 degrees). Participants arranged eight different sets of cards, four sets in each sitting. Testing was conducted midday or early afternoon outside on the front porch of a house in the shade. Participants were tested by SF in Mian with occasional further explanations in Tok Pisin. A complete set of experimental materials as well as a detailed description of methods, procedures, and instructions is available in Boroditsky et al. (2007).

### Data coding

Each participant’s arrangement was diagrammed by the experimenter. The sessions were also video-recorded. The arrangements were then coded in both absolute and relative spatial coordinates by two naïve coders, unaware of the purpose of the study. We used cardinal directions for coding (rather than Mian river directions) to allow for ease of comparison and aggregation with studies conducted at other sites. The codings were quantified by assigning each of the four main directions (within a coordinate frame) one of five possible values (0, 0.25, 0.5, 0.75, or 1), with the sum of the four directions adding up to 1. Some example codings: if the arrangement was laid out from north to south, the directionality coding for that trial would be *N* = 0, *E* = 0, *S* = 1, *W* = 0. If the arrangement was toward the NW, the directionality coding would be *N* = 0.5, *E* = 0, *S* = 0, *W* = 0.5. If the arrangement was toward the ESE, the directionality coding for that trial would be *N* = 0, *E* = 0.75, *S* = 0.25, *W* = 0. To obtain summary statistics, we computed the average value for each of the four main directions in each coordinate frame (*N*/*S*/*E*/*W* in absolute coordinates and Left/Right/Toward/Away in relative space).

We also converted these two-dimensional axes-based representations into degrees around the compass (by computing the arc-tangent between the values on the two axes, adjusting any negative radian values by adding 2π and converting into degrees). In this coding, an arrangement that went from south to north was coded as 0 degrees, an arrangement from east to west was coded as 90 degrees and so on. All of the produced arrangements were deemed to be interpretable as having a linear order, and so all arrangements were included in the analysis. The codings produced by the two independent coders were on average within 33 degrees of each other and revealed the same overall pattern. Discrepancies were resolved upon discussion and consultation with the field experimenter (SF).

### Results

Of the nine participants tested, six showed a body-relative pattern when laying out time, and three showed an absolute spatial pattern.

Four participants produced a consistent left to right relative pattern (average directionality was 0.99 left to right). That is, they laid out the cards such that time progressed from left to right with respect to their bodies, regardless of their cardinal facing direction. This is the same pattern as generally seen in American English speakers.

Two participants arranged time along the sagittal axis, with cards showing earlier events further away from the body and cards showing later events placed closer to the body (average directionality was 0.90 toward the body). These two participants again used this toward the body arrangement regardless of their cardinal facing orientation.

Finally, three participants consistently produced temporal arrangements that were oriented in absolute space (they had different orientation with respect to the body, depending on the participant’s cardinal facing direction). All three arranged the cards primarily along the east-west axis. Two of the participants laid out time as proceeding from east to west (average compass angle for later events = 276 degrees), and one participant laid out time as proceeding from west to east (average compass angle for later events = 100 degrees).

The absolute arrangements appear to be rotated slightly (Mean = 7 degrees) clockwise off of the east-west axis. One possible explanation for such a rotation may relate to the direction of the river. The rivers in this region flow to the WNW. It is possible that participants intended to arrange time as going upriver or downriver rather than on the east-west axis *per se*. Another possibility is that the participants intended to arrange time along the east-west axis but that the direction of the river has coerced people’s representations of east and west.

We analyzed the participants’ arrangement types (left to right, toward the body, or absolute) as a function of age, education level, and literacy. For each time orientation, we coded a participant as a 1 if that was the dominant orientation of their responses and a 0 if it was not the dominant orientation. We then computed by-participants (*N* = 9, df = 7) Pearson correlations within each time orientation to determine whether individual differences in education or age can be used to predict individual differences in time orientation.

From these analyses, only the number of years of formal education emerged as a significant predictor of temporal arrangement type. Greater number of years of formal education positively predicted left to right arrangements [*r*(7) = 0.61, *p* < 0.05] and negatively predicted absolute spatial arrangements [*r*(7) = −0.65, *p* < 0.05]. No other factors emerged as statistically reliable. Because the number of participants in our study is small (an unfortunate field-site limitation), this analysis is best treated as a preliminary observation. A larger sample would be necessary to establish generality and tease apart more fine-grained relationships.

### Discussion

The overall pattern of results reveals a variety of representational strategies for organizing time among the Mianmin. In addition to the left to right pattern seen with North American English speakers, the Mianmin also produced consistent body-relative patterns that oriented time as coming toward the body. Importantly, a third of the participants did not lay out time with respect to the body, but instead arranged it roughly along the east-west axis in absolute space. The variability in time arrangements observed even in our small sample suggests that the spatialization of time among the Mianmin is less standardized than it is in industrialized Western cultures, with a variety of representations readily cognitively available.

The absolute pattern differs strikingly from patterns observed on such tasks previously with speakers of English, Mandarin, Arabic, and Hebrew (e.g., Tversky et al., 1991; Chan and Bergen, 2005; Fuhrman and Boroditsky, 2010). Such a pattern has been observed previously in Kuuk Thayorre speakers of the Australian Aboriginal community of Pormpuraaw, where absolute spatial frames of reference are favored over relative terms like left and right in the local languages for describing space (Boroditsky and Gaby, 2010).

These data from Mian suggest that absolute patterns of laying out time are more broadly distributed around the world. The Mianmin live in a very different physical environment than that of Pormpuraaw. Pormpuraaw is an expansive largely flat environment, bounded by the open ocean. The Mianmin live in a rugged and mountainous region covered with primary and secondary rain forest. The Pormpuraawans are hunter-gatherers, while the Mianmin are subsistence farmers. The existence of absolute representations of time among the Mianmin suggest that absolute spatial representations of time are not restricted to a particular geographical location, particular type of physical environment, or particular lifestyle. What the two communities do share is that in both, the spoken languages rely heavily on absolute spatial frames of reference when talking about space. Using such languages requires one to stay oriented in one’s environment, in order to be able to speak the language properly. It appears that when representations of space with respect to the landscape (as opposed to with respect to the body) become culturally salient, people are also likely to create representations of time as laid out on the landscape. Further, it appears that the absolute spatial patterns of organizing time are weakened with more exposure to formal education, in favor of left to right representations, which are ubiquitous in western educational settings. Further research is needed to explore the generalizability of this relationship to larger samples and other communities.

Arrangements of time as coming toward the body might be related to the metaphorical extensions of the upstream-downstream properties of the river to personal space, where “upriver” is near the speaker and “downriver” is away from the speaker. If the participants saw the task as invoking a communicative frame and interpreted the picture cards as a story being told to them, this may explain the tendency to arrange time as coming toward the body (or metaphorically downriver toward the addressee). Of course, further work is necessary to test this possibility.

What can explain the east/west axis pattern found in one third of the participants? There are two possibilities: the motion of the sun, and the orientation of the river. The river is oriented roughly along the east-west axis, rotated slightly clockwise off of the compass axis. On average, the axis of the absolute temporal arrangements produced was rotated slightly clockwise off of the east-west compass axis. It is possible that the Mianmin showed absolute patterns of organizing time according to the direction of the river. Another possibility is that the direction of the river coerces people’s representations of east and west, such that participants meant to organize time according to the axis of the motion of the sun, but their representation of this axis is rotated slightly to match the orientation of the river. Future work can help disentangle these two possibilities, for example by testing at locations where the river turns and takes a different direction.

## Conclusion

We examined representations of time among the Mianmin of Papua New Guinea. First, we described the patterns of spatial and temporal reference in Mian, which uses a system of spatial terms that derive from the orientation and direction of the Hak and Sek rivers and the surrounding landscape. We also examined how the Mianmin spatialize time in a non-linguistic temporal reasoning task. The results revealed a variety of temporal representations. Some participants arranged time with respect to their bodies (left to right or toward the body). Others arranged time as laid out on the landscape, roughly along the east/west axis, consistent both with the axis of the motion of the sun and the orientation of the two rivers (which provide the basis for spatial reference in Mian). Our data also provided an initial indication for the role of formal schooling: participants with more formal education were more likely to arrange time from left to right (the dominant pattern found in American English speakers), while participants with less formal education were more likely to produce an absolute representation of time, roughly along the east-west axis (a pattern not found with American English speakers, but observed in other communities that rely on absolute spatial frames of reference). Further work with larger samples is needed to further examine this relationship. The results of our study extend previous work on spatial representations for time to a new geographical region, physical environment, and linguistic and cultural system.

## Conflict of Interest Statement

The authors declare that the research was conducted in the absence of any commercial or financial relationships that could be construed as a potential conflict of interest.

## Footnotes

^1^An expedition was launched between the Fly and Sepik headwaters in the mid-1920s (Champion, 1966) and then mining investigations were carried out in the mid-1930s (Kienzle and Campbell, 1938), during which first contact with the Mianmin was established (Campbell, 1938: 245).

^2^Heine also notes that the front of trees that are perceived as completely vertical is assigned to the direction of the biggest branch or the location with the most branches. We have no parallel information for Mian about the assignment of the back in absolutely vertical trees.

^3^The superscript “H” indicates that many forms inflected for the “Non-hodiernal past” bear a high tone on the subject suffix, which follows the tense suffix. Although the appearance of the high tone is irregular and does not appear in all Non-hodiernal past forms I include the tonal specification to distinguish *-b^H^* “Non-hodiernal past” from *-b* “Imperfective.”

^4^The traditional view of the world held that the people lived on *dabáal* “earth, ground,” which was surrounded by a saltwater ocean *(amúk sum*, literally “big lake”). Only initiated man were privy to this knowledge. The sun moved in circles above (day) and below (night) the earth and the surrounding ocean, which were both stationary. The moon moved toward (full moon) and away from (new moon) the earth. It was believed that the moon had its own place to which it returned once every month at new moon. The waxing and waning of the moon was cast in a metaphor: “His way is like that of children, ever growing.” The stars were called the “light of the night.” They were stationary and of little mythical significance, except that they, together with the moon, contain *akgit* “dew,” which they put on the earth to make things grow. Nowadays, the views of modern science have been adopted.

^5^All degrees of language proficiency and literacy were assessed by the experimenter. Language proficiency was assessed on a scale from 1 to 5, literacy was assessed on a scale from 0 to 10.

## Abbreviations

1, first person; 2, second person; 3, third person; an, animate; aux, auxiliary; decl, declarative; ds, different subject; emph, emphatic; func, functional verb; ipfv, imperfective; loc, locative; m, masculine; med, medial; n1, neuter 1; n2, neuter 2; o, object; pfv, perfective; pl, plural; pn, proper name; real, realis; sbj, subject; seq, sequential; sg, singular; ss, same subject; vblz, verbalizer.
